# ﻿The genus *Pycnolejeunea* (Lejeuneaceae, Marchantiophyta) in Thailand, with the description of *Pycnolejeuneazhuiana*

**DOI:** 10.3897/phytokeys.259.156710

**Published:** 2025-07-01

**Authors:** Chatchaba Promma, Sahut Chantanaorrapint

**Affiliations:** 1 PSU-Herbarium, Division of Biological Science, Faculty of Science, Prince of Songkla University, Hat Yai, Songkhla 90110, Thailand Prince of Songkla University Songkhla Thailand

**Keywords:** Endemic species, leafy liverwort, ocellus, Peninsular Thailand, subtribe Pycnolejeuneinae

## Abstract

A taxonomic study of the genus *Pycnolejeunea* in Thailand is presented, based on herbarium specimens and new collections from field surveys. Three species are recognised including *P.contigua*, *P.grandiocellata* and one new species, *P.zhuiana*. The new species is distinguished by the strongly papillose lobules, the presence of 1–8 basal ocelli on the lateral leaf and male bracteoles occurring along nearly the whole of the androecial branch length. A key to species, descriptions and illustrations are provided; ecology and geographical distribution of the species are also described.

## ﻿Introduction

The genus *Pycnolejeunea* (Spruce) Schiffn. was originally described by Spruce (1884) as a subgenus of *Lejeunea* Libert. and was raised to generic rank by Schiffner (1893). The genus *Pycnolejeunea* was monographed by He (1999), who accepted nine species worldwide. In the world checklist of hornworts and liverworts (Söderström et al. 2016), 21 species were accepted, including seven doubtful species. Subsequently, three additional species were described (Bastos and Zartman 2017; Reiner-Drehwald and Gradstein 2018; Thouvenot and Gradstein 2021), increasing the total number of species to 24. *Pycnolejeunea* is a pantropical genus, with the greatest diversity found in the Neotropics, where ten species are known to occur (Reiner-Drehwald and Gradstein 2018; Bastos et al. 2020). Species of *Pycnolejeunea* are corticolous epiphytes growing on tree trunks and twigs in tropical lowland and submontane rainforests. *Pycnolejeunea* can be recognised by the following morphological characteristics: 1) rigid stems composed of thick-walled cells; 2) imbricate and convex leaf lobes; 3) leaf lobe cells being mammillose or papillose, rarely plane, with distinct and rather large trigones; 4) the presence of ocelli in leaf lobes and female bracts; 5) large coarsely granular oil bodies; 6) lobules featuring a well-developed first tooth with a marginal hyaline papilla; 7) gynoecia with pycnolejeuneoid innovations and 8) perianths with five smooth to slightly crenate keels. *Pycnolejeunea* might be easily confused with *Cheilolejeunea* (Spruce) Steph. in general appearance. However, *Cheilolejeunea* is distinguished by the elaborated second tooth, which causes the papilla to appear distal (in *Pycnolejeunea*, the second tooth is small compared to the first tooth and the papilla appears proximal) and usual lack of ocelli (Gradstein 2021).

In Thailand, *Pycnolejeunea* was first documented by Stephani (1902), who described a new species, *Pycnolejeuneagrandiocellata* Steph., from Koh Chang, Trat Province. More than 100 years later, *P.contigua* (Nees) Grolle was reported from coastal sand dune forest, Phang Nga Province (Suwanmala and Chantanaorrapint 2016). More recently, Senayai et al. (2020) discovered three *Pycnolejeunea* species including: *P.contigua*, *P.grandiocellata* and *P.cavistipula* (Steph.) Mizut. from Khao Ngon Nak Mountain, Hat Noppharat Thara - Mu Ko Phi Phi National Park, Krabi Province. After re-examination of the specimens of *P.cavistipula*, they were misidentified and resemble *P.papillosa* X.-L. He from tropical America in having papillose lobules (He 1999). Following a detailed comparison with closely-related taxa, we here describe these specimens as a new species. The aim of the present study was to revise the genus *Pycnolejeunea* in preparation for the Bryophyte flora of Thailand.

## ﻿Materials and methods

This study is based on recent collections from Thailand as well as herbarium specimens housed in BKF, EGR and PSU. Morphological and anatomical characters were studied using stereo- and compound microscopes. The distinctive characters of the species were photographed by an Olympus BX51 light microscope with attached Olympus DP74 Microscope Digital Camera and illustrated with Nikon Eclipse E200 with attached Nikon Y-IDT Drawing Tube after fully rehydrating samples with tap water. The distinctive characters of the new species were examined and photographed by an FEI Quanta 400 scanning electron microscope. Voucher specimens of the new species are deposited in BKF, NICH and PSU Herbaria. Descriptions, illustrations and a key to species of the genus *Pycnolejeunea* in Thailand are provided. In addition, distribution and ecological data were compiled.

## ﻿Taxonomic treatments

### ﻿Key to species of the genus *Pycnolejeunea* in Thailand

**Table:** 

1	Lobule cells strongly papillose; leaf cells strongly mammillose on the dorsal side	***P.zhuiana***
–	Lobule cells nearly smooth or slightly convex; leaf cells slightly convex or weakly mammillose on the dorsal side	**2**
2	Ocelli suprabasal, mostly 4–15 per leaf lobe, commonly aggregated; lobule oblong to rectangular; plants without flagelliform shoots	***P.grandiocellata***
–	Ocelli basal, mostly 1–5 per leaf lobe, isolated or aggregated; lobule ovate; plants usually with flagelliform shoots	***P.contigua***

#### ﻿1. Pycnolejeunea contigua

Taxon classificationPlantaeLejeunealesLejeuneaceae

(Nees) Grolle, J. Hattori Bot. Lab. 45: 179. 1979.

88F3FD8C-8437-5B1A-876B-B9D3797EDBA3

Figs 1 , 2

≡ Jungermanniacontigua Nees in Martius, Fl. Brasil. enum. plant.1(2): 360. 1833. Type: Brazil. Pará: ad corticem arborum, *C.F. Martius s.n.* (isotype: G [G00128260]).
= Pycnolejeuneabancana Steph., Hedwigia 35: 124. 1896. Type: Indonesia. Insula Banca, 1883, *J.E. Teysmann s.n.* (lectotype: G [G00281813], designated by He (1999)).
= Pycnolejeuneapapulosa Steph., Hedwigia 35: 125. 1896. Type: Brazil. Pará: Caripi, *R. Spruce s.n.* (lectotype: G [G00128263], designated by Grolle (1979); isolectotypes: G [G00128261, G00128262], JE, M, W).
= Pycnolejeuneadensiuscula Spruce ex Steph., Sp. Hepat. 5: 613. 1914. Type: Brazil. Pará: Silva Amazonica, Santarém, Dec 1849, *R. Spruce s.n.* (lectotype: G [G00128259] designated by Grolle (1979); isotype: G [G00128258], JE, M).
= Pycnolejeuneaocellata Steph., Sp. Hepat. 5: 614. 1914. Type: Cuba. *C. Wright s.n.* (lectotype: G [G00128226], designated by He (1999); isolectotype: JE [JE04002710]).

##### Description.

***Plants*** whitish-green or light green when fresh, yellowish-brown or light brown in dry condition; shoots 0.9–1.5 mm wide, usually scarcely and irregularly branched; branches *Lejeunea*-type. ***Stems*** 100–130 µm diameter, in transverse section with 9(–10) epidermal cells, surrounding 14–18 medullary cells, epidermal cells larger than medullary cells; cell walls pale brown or yellowish-brown, thick-walled, with triangular to bulging trigones, wall between trigones with thin to rather thick continuous thickenings; ventral merophyte 2 cells wide. ***Rhizoids*** at base of underleaves, few, tufted, usually hyaline, rhizoid disc not seen. ***Leaves*** closely imbricate, when moist, wide-spreading. ***Leaf lobes*** ovate to oblong-ovate, rarely falcate-ovate, 725–827 µm long, 544–617 µm wide, dorsal margin arched, ventral margin arched, margin entire to slightly crenulate with projecting cells, apex rounded, incurved. Lobe cells convex or weakly mammillose on dorsal side, thin-walled, with small to large triangular trigones, intermediate thickenings absent or occasionally seen in the basal cells; marginal cells rectangular or quadrate, 16.5–20.8 × 15.0–23.7 µm; median cells hexagonal to rounded, 22.5–37.0 × 21.5–38.5 µm, basal cells hexagonal to rectangular, 22.0–45.8 × 20.0–30.0 µm; ocelli rectangular to long hexagonal, 47–65 × 25–40 µm, (0–)1–5 per leaf lobe, basal, aggregated or isolated; oil bodies 3–5 per cell, long ellipsoidal to ovoid-cylindrical, 10.4–19.8 × 3.7–5.1 µm, *Calypogeia*-type, coarsely granular. ***Lobules*** small, ovate, 152–182 µm long, 120–135 µm wide, inflated, 0.2–0.3 of lobe length, free margin slightly involute, formed by 5–7 elongated cells, apex semicircular, apical tooth short, 1-celled, obtuse, keel arched or nearly straight, lobule cells smooth or slightly convex. ***Underleaves*** imbricate, rarely contiguous, suborbicular to reniform, 287–330 µm long, 416–458 µm wide, wider than long, 3–5 of stem width, bifid to 1/2 of its length, lobes triangular with acute to obtuse apex, margin nearly entire or bluntly toothed at side, sinus V-shaped, bases rounded to cuneate, insertion line arched. ***Asexual reproduction*** by unmodified caducous leaves, lobules remain attached to the stem; or modified caducous leaves arising from upright flagelliform shoots on branch apices, smaller than ordinary leaves, margins usually with 1-celled rhizoids. ***Sexuality*** autoicous. ***Androecia, gynoecia and sporophytes*** not seen.

**Figure 1.**
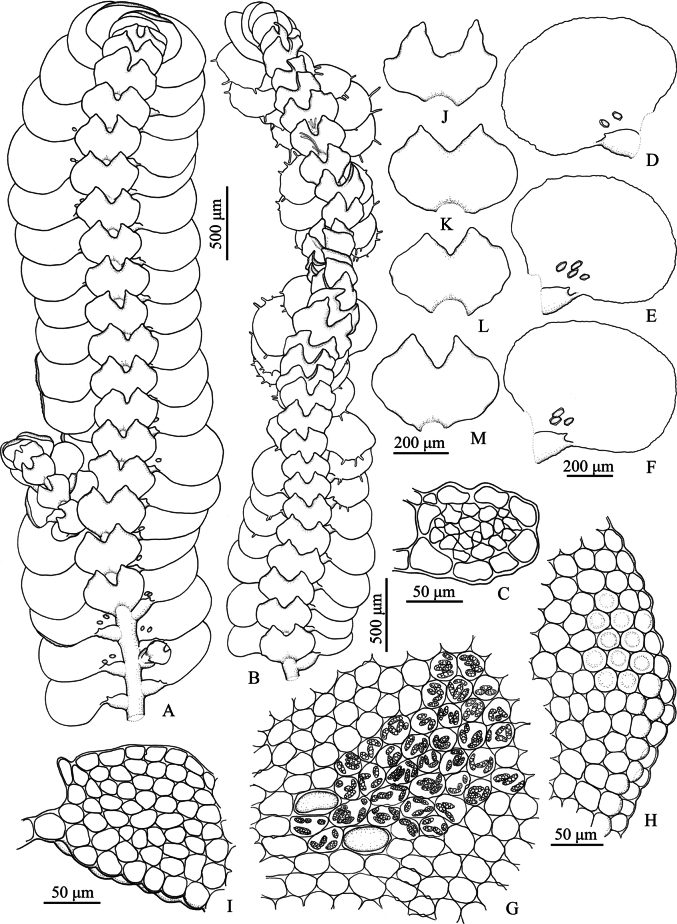
*Pycnolejeuneacontigua* (Nees) Grolle. **A.** Portion of sterile plant, ventral view; **B.** Portion of sterile plant with flagelliform shoot, ventral view; **C.** Transverse section of stem; **D–F.** Lateral leaves; **G.** Cells from basal and near middle portion of leaf, dorsal view; **H.** Cells from apical leaf margin; **I.** Leaf lobule; **J–M.** Underleaves. Drawn by C. Promma; based on *C. Promma & K. Chanakarn 20250215-28B* (PSU).

**Figure 2.**
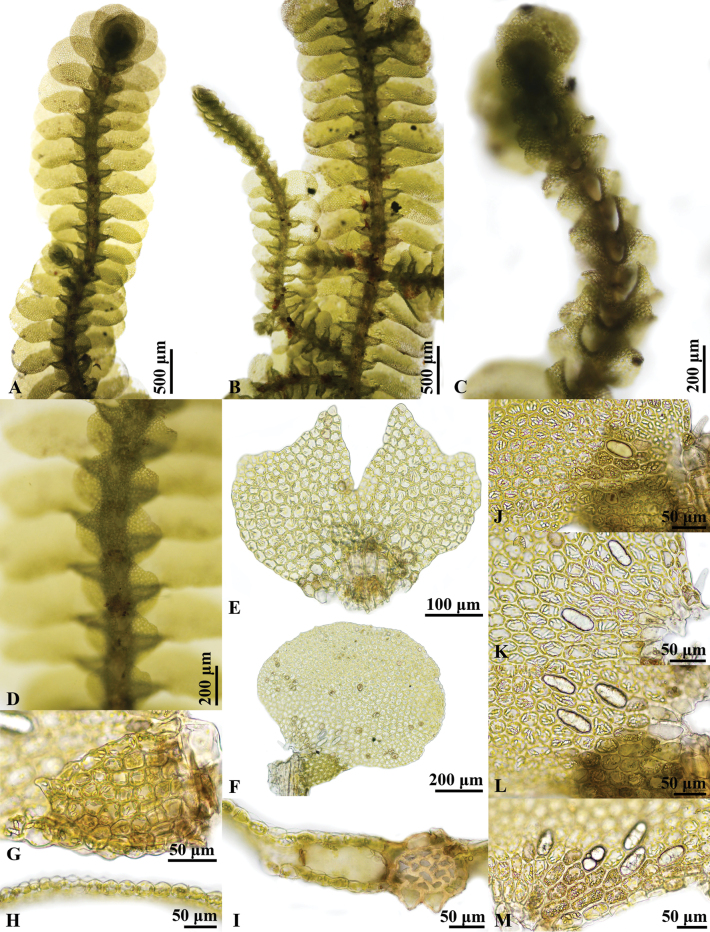
*Pycnolejeuneacontigua* (Nees) Grolle. **A.** Portion of sterile plant, ventral view; **B.** Portion of sterile plant with flagelliform shoot, ventral view; **C.** Flagelliform shoot; **D.** Portion of sterile plant, showing underleaves; **E.** Underleaf; **F.** Lateral leaf; **G.** Leaf lobule; **H.** Transverse section of leaf; **I.** Transverse section of stem; **J–M.** Cells from basal portion of leaf, showing ocelli. Photographed by C. Promma; based on *C. Promma & K. Chanakarn 20250215-28B* (PSU).

##### Distribution, habitat and ecology.

Pantropical (He 1999; Bastos et al. 2020). In Thailand, *Pycnolejeuneacontigua* was found on tree trunks and branches in forest gaps, along forest margins or open habitats in coastal sand dune and tropical lowland forests, ranging from sea level to 480 m above sea level.

##### Taxonomic notes.

*Pycnolejeuneacontigua* is a widely distributed species distinguished by its pale to glossy brownish plants, 1–5 basal ocelli per leaf lobe (aggregated or isolated) and large broadly ovate to reniform underleaves that almost completely cover the lobules. Asexual reproduction occurs via unmodified caducous leaves with lobules remaining attached to the stem and usually by modified caducous leaves arising from flagelliform shoots.

In Thailand, *P.contigua* is most similar to *P.grandiocellata*, sharing several vegetative features such as ovate to oblong leaf lobes, 2-celled wide ventral merophytes and smooth to slightly convex lobule cells. However, *P.contigua* differs in having 1–5 ocelli which are basal in position, whereas *P.grandiocellata* exhibits more ocelli (4–15) suprabasal ocelli commonly aggregated from the base to the ventral half of the lobe. Additionally, the presence of modified caducous leaves (flagelliform shoots) in *P.contigua* further distinguishes it from *P.grandiocellata*, which produces only unmodified caducous leaves.

##### Specimens examined.

Thailand. Phang Nga: Hat Thai Mueang - Khao Lampi National Park, 8°29.1523'N, 98°13.6872'E, 18 m elev., 10 Oct 2015, *O. Suwanmala 111, 119A* (PSU); 8°29.011'N, 98°13.7605'E, 13 m elev., 30 Jan 2016, *O. Suwanmala 186* (PSU); Krabi: Hat Noppharat Thara - Mu Ko Phi Phi National Park, Khao Ngon Nak Mountain, 8°5.2767'N, 98°46.47'E, 480 m elev., 24 Mar 2018, *A. Senayai 75a, 126b* (BKF, PSU); 12 Oct 2018, *A. Senayai 212* (BKF, PSU); 8°5.2767'N, 98°46.47'E, 480 m elev., 15 Feb 2025; *C. Promma & K. Chanakarn 20250215-19, 20250215-28B* (PSU); Yala: Betong, Ban Piyamit 2, 14 Jun 2013, *S. Chantanaorrapint & C. Promma 2515* (PSU).

#### ﻿2. Pycnolejeunea grandiocellata

Taxon classificationPlantaeLejeunealesLejeuneaceae

Steph. in Schmidt, Fl. Koh Chang, Bot. Tidsskr. 24: 279. 1902.

5BFF33BB-3C9E-53E0-A0F3-61966E331D60

Figs 3 , 4

##### Type material.

Thailand. Trat: Klong Munse, 1899–1900, *E. J. Schmidt 6* (holotype: G [G00128271]).

##### Description.

***Plants*** whitish-green or light green when fresh, yellowish-brown or light brown in dry condition; shoots 0.80–1.23 mm wide, usually scarcely and irregularly branched; branches *Lejeunea*-type. ***Stems*** 76–99 µm diameter, in transverse section with 8–11 epidermal cells, surrounding 9–14 medullary cells, epidermal cells larger than medullary cells; cell walls pale brown or yellowish-brown, thick-walled, with triangular to bulging trigones, wall between trigones with thin to rather thick continuous thickenings; ventral merophyte 2 cells wide. ***Rhizoids*** at base of underleaves, few, tufted, usually hyaline, rhizoid disc not seen. ***Leaves*** imbricate, wide-spreading when moist. ***Leaf lobes*** ovate, oblong-ovate to oblong, 468–680 µm long, 376–487 µm wide, dorsal margin broadly arched, ventral margin slightly to strongly arched, margin entire, apex rounded, incurved. Lobe cells convex or weakly mammillose on dorsal side, thin-walled, with small to large triangular trigones, intermediate thickenings absent or occasionally seen in the marginal and basal cells; marginal cells rectangular or quadrate, 18.0–22.8 × 11.3–23.0 µm, median cells hexagonal to rounded, 18.0–38.3 × 19–24.8 µm, basal cells hexagonal to rectangular, 25.0–41.5 × 20.0–26.0 µm; ocelli rectangular to long hexagonal, 39.5–63.5 × 21.0–36.5 µm, 4–15 per leaf lobe, suprabasal, commonly aggregated, confined from base to the ventral half of leaf lobe; oil bodies not seen. ***Lobules*** small, oblong, 132–178 µm long, 90–109 µm wide, inflated, 0.16–0.30 of lobe length, free margin slightly involute, formed by 5–7(–9) elongated cells, apex semicircular or rarely truncate, apical tooth short, 1-celled, obtuse, keel arched or nearly straight, lobule cells smooth or slightly convex. ***Underleaves*** contiguous to imbricate, sometimes slightly remote, suborbicular to subreniform, 116–297 µm long, 206–363 µm wide, wider than long, ca. 3 of stem width, bifid to 1/3–1/2 of its length, lobes triangular with acute to obtuse apex, margin nearly entire or bluntly toothed at side, sinus V-shaped, bases rounded to cuneate, insertion line arched. ***Asexual reproduction*** by unmodified caducous leaves, lobule remain attached to the stem. ***Sexuality*** autoicous. ***Androecia*** on short branches, terminal or intercalary on branches, inflated, spicate, 407–663 µm long, 452–607 µm wide; bracts in 2–4 pairs, densely imbricate, isolobous; bracteole 1–2, restricted at the base of the branch, bifid. ***Gynoecia*** on short branches, with 1(–2) subfloral innovations; bracts in one pair, subequal in size, bract lobes obovate, 733–829 µm long, 286–563 µm wide, apex rounded, incurved, margin entire; ocelli 10–26 per lobe or numerous, aggregated from base to the middle of the lobe; bract lobules lingulate to narrowly oblong, 449–503 µm long, 126–157 µm wide, 0.5–0.8 of lobe length, ca. 2/3 of the bract-lobe area, apex acute to obtuse, keel slightly arched, short; bracteole shortly connate with the bracts at the base on one side or rarely on both sides, ovate to obovate, 479–602 µm long, 253–295 µm wide, apex usually emarginate or slightly bifid, lobe acute, margin entire; perianths obovate, ca. 0.5 emergent beyond bracts or sometimes almost entirely covered by bracts, 687–825 µm long, 380–488 µm wide, inflated, 5-keeled, keels smooth or crenulate, apex usually truncate, beak short. ***Seta*** articulate. ***Capsule*** valves 4, broadly spreading after dehiscence. ***Elaters*** 30 per capsule, marginal elaters 22, upper ends attached to valve margins, inner elaters 8, usually rudimentary, both ends attached to valve surface. **Spores** not seen.

**Figure 3.**
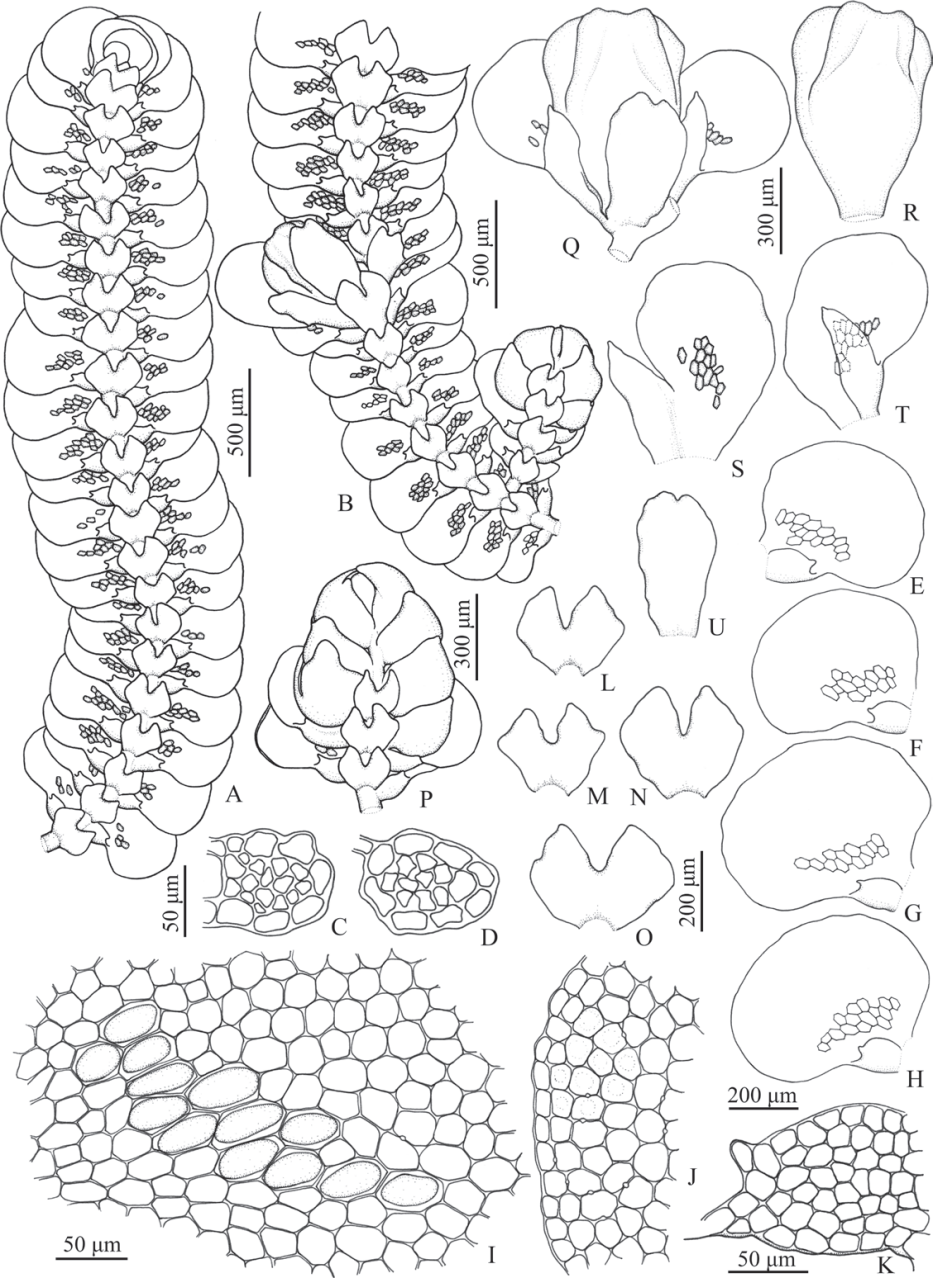
*Pycnolejeuneagrandiocellata* Steph. **A.** Portion of sterile plant, ventral view; **B.** Portion of fertile plant, ventral view; **C, D.** Transverse sections of stems; **E–H.** Lateral leaves; **I.** Cells from basal and near middle portion of leaf, dorsal view; **J.** Cells from apical leaf margin; **K.** Leaf lobule; **L–O.** Underleaves; **P.** Androecium; **Q.** Gynoecium; **R.** Perianth; **S, T.** Female bracts; **U.** Female bracteole. Drawn by C. Promma; based on *T. Pócs & S. Somadee 1227/K* (PSU).

**Figure 4.**
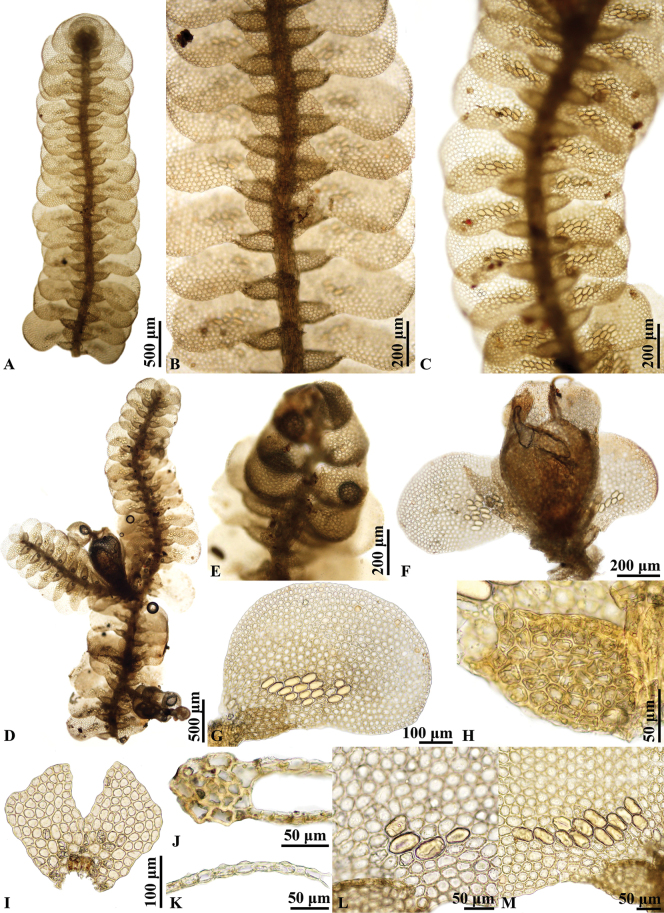
*Pycnolejeuneagrandiocellata* Steph. **A–C.** Portions of sterile plants, ventral view; **B.** Showing underleaves; **C.** Showing ocelli; **D.** Portion of fertile plant, ventral view; **E.** Androecium; **F.** Gynoecium; **G.** Lateral leaf; **H.** Leaf lobule; **I.** Underleaf; **J.** Transverse sections of stem; **K.** Transverse section of leaf; **L, M.** Cells from basal portion of leaf, showing ocelli. Photographed by C. Promma; based on *T. Pócs & S. Somadee 1227/K* (PSU).

##### Distribution, habitat and ecology.

Tropical Asia, Australia and Oceanic Islands (He 1999; Yang and Lin 2011). In Thailand, *Pycnolejeuneagrandiocellata* was found growing on tree trunks and branches in tropical lowland and submontane forests at 65–1200 m in elevation.

##### Taxonomic notes.

*Pycnolejeuneagrandiocellata* is easily recognized by a combination of the following characters: 2 cells wide ventral merophytes, closely imbricate leaf lobes, numerous suprabasal ocelli which are commonly aggregated and the oblong leaf lobule with 5–7 elongated cells along the free margin. *Pycnolejeuneagrandiocellata* resembles *P.contigua* in general appearance. For distinguishing characters of these two species, refer to the taxonomic notes section of *P.contigua*.

##### Specimens examined.

Thailand. Krabi: Hat Noppharat Thara - Mu Ko Phi Phi National Park, Khao Ngon Nak Mountain, 8°5.8267'N, 98°45.1067'E, 150 m elev., 24 Mar 2018, *A. Senayai 21* (BKF, PSU); 8°5.415'N, 98°46.1683'E, 380 m elev., 24 Mar 2018, *A. Senayai 28a* (BKF, PSU); 13 Oct 2018, *A. Senayai 337b* (BKF, PSU); 8°5.4'N, 98°46.1517'E, 452 m elev., 18 Feb 2019, *A. Senayai 411* (BKF, PSU); 8°5.4'N, 98°46.1517'E, 456 m elev., 12 Jun 2019, *A. Senayai 416* (BKF, PSU); *A. Senayai 417*, *418a* (BKF, PSU); 8°5.8267'N, 98°45.1067'E, 96 m elev., 18 Feb 2019, *A. Senayai 506a* (BKF, PSU); Klong Thom, Sa Morakot (Emerald Pool), 65 m elev., 9 Nov 2012, *T. Pócs & S. Somadee 1227/K* (EGR, PSU).

#### ﻿3. Pycnolejeunea zhuiana

Taxon classificationPlantaeLejeunealesLejeuneaceae

Promma & Chantanaorr. sp. nov.

89C57BD5-CCB9-501A-856F-0CE66FBC350A

Figs 5 , 6 , 7

##### Type material.

Thailand. Krabi: Hat Noppharat Thara - Mu Ko Phi Phi National Park, Khao Ngon Nak Mountain, 8°5.2767'N, 98°46.47'E, 480 m elev., 15 Feb 2025, *C. Promma & K. Chanakarn 20250215-30* (holotype: PSU!; isotypes: BKF!, NICH!).

**Figure 5.**
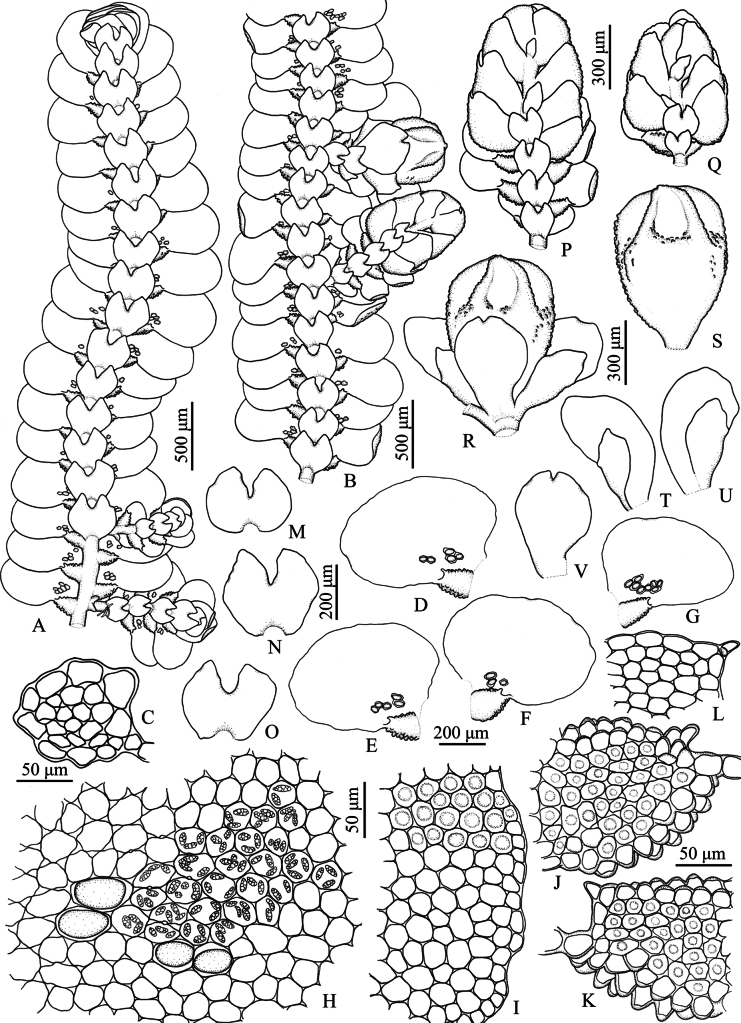
*Pycnolejeuneazhuiana* Promma & Chantanaorr. sp. nov. **A.** Portion of sterile plant, ventral view; **B.** Portion of fertile plant, ventral view; **C.** Transverse sections of stem; **D–G.** Lateral leaves; **H.** Cells from basal and near middle portion of leaf, dorsal view; **I.** Cells from apical leaf margin; **J–L.** Leaf lobules; **L.** Leaf lobule from inner side with hyaline papilla; **M–O.** Underleaves; **P, Q.** Androecia; **R.** Gynoecium; **S.** Perianth; **T, U.** Female bracts; **V.** Female bracteole. Drawn by C. Promma; based on *C. Promma & K. Chanakarn 20250215-30* (PSU).

##### Diagnosis.

*Pycnolejeuneazhuiana* similar to *P.papillosa*, but differs in having 1–8 ocelli per leaf lobe, stems in transverse section composed of 9–11 epidermal cells surrounding 13–15 medullary cells and male bracteoles occurring along nearly the whole of the androecial branch length.

**Figure 6.**
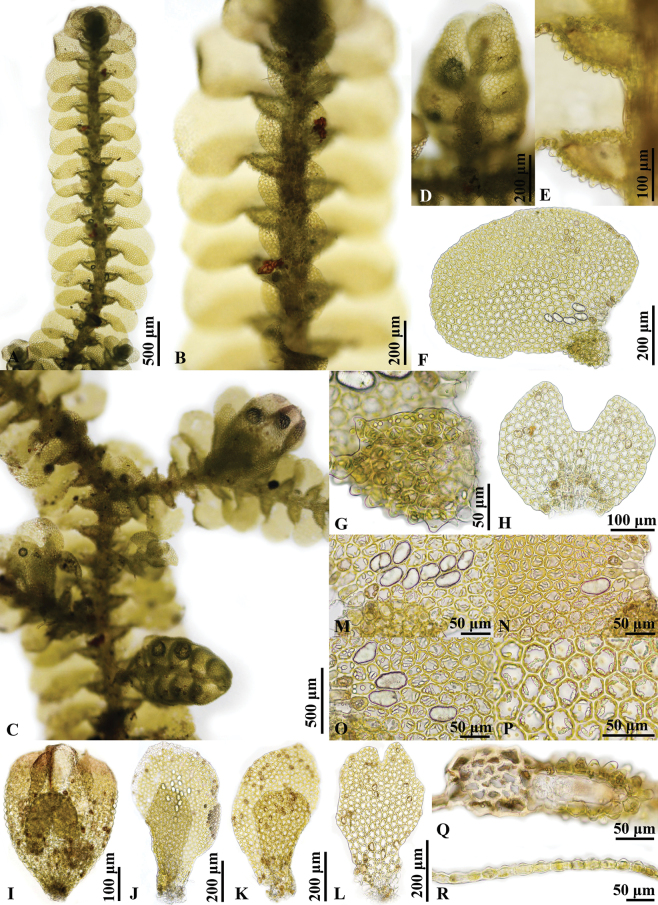
*Pycnolejeuneazhuiana* Promma & Chantanaorr. sp. nov. **A, B.** Portions of sterile plants, ventral view; **B.** Showing underleaves; **C.** Portion of fertile plant, ventral view; **D.** Androecium; **E.** Portions of sterile plants, showing lobule; **F.** Lateral leaf; **G.** Leaf lobule; **H.** Underleaf; **I.** Perianth; **J, K.** Female bracts; **L.** Female bracteole; **M–O.** Cells from basal portion of leaf, showing ocelli; **P.** Cells from middle portion of leaf, ventral view; **Q.** Transverse sections of stem and lobule; **R.** Transverse section of leaf. Photographed by C. Promma; based on *C. Promma & K. Chanakarn 20250215-30* (PSU).

##### Description.

***Plants*** whitish-green or light green when fresh, yellowish-brown or light brown in dry condition; shoots with leaves 0.71–1.32 mm wide; usually scarcely and irregularly branched; branches *Lejeunea*-type. ***Stems*** 117–132 µm diameter, in transverse section with 9–11 epidermal cells, surrounding 13–15 medullary cells, epidermal cells larger than medullary cells; cell walls pale brown or yellowish-brown, thick-walled, with triangular to bulging trigones, wall between trigones with thin to rather thick continuous thickenings; ventral merophyte 2 cells wide. ***Rhizoids*** at base of underleaves, few, tufted, usually hyaline, rhizoid disc not seen. ***Leaves*** imbricate, wide-spreading when moist. ***Leaf lobes*** ovate to oblong-ovate, occasionally falcate-ovate, 563–698 µm long, 429–512 µm wide, dorsal margin broadly arched, ventral margin slightly to strongly arched, margin entire or conspicuously crenulate with projecting cells, apex rounded, incurved. Lobe cells strongly mammillose on dorsal side, thin-walled, with small to large triangular trigones, intermediate thickenings absent or occasionally seen in the basal cells; marginal cells rectangular or quadrate, 12.5–20.5 × 12–18 µm, median cells hexagonal to rounded, 20.5–34.0 × 19.0–27.5 µm, basal cells hexagonal to rectangular, 22.0–42.6 × 20.0–30.5 µm; ocelli rectangular to long hexagonal, 35.0–61.7 × 23.6–36.0 µm, 1–8 per leaf lobe, basal, aggregated or isolated; oil bodies 2–5 per cell, long ellipsoidal to ovoid-cylindrical, 7.0–19.0 × 4.0–7.0 µm, *Calypogeia*-type, coarsely granular. ***Lobule*** small, ovate, 138–170 µm long, 125–156 µm wide, strongly inflated, 0.20–0.25 of lobe length, free margin slightly involute, formed by 5–6 elongated cells, apex semicircular, apical tooth short, obtuse; keel strongly arched, papillose; lobule cells strongly unipapillose. ***Underleaves*** contiguous to imbricate, sometimes slightly remote, suborbicular to subreniform, 234–316 µm long, 257–360 µm wide, wider than long, 3.0–3.5 of stem width, bifid to 1/3–1/2 of its length, lobes triangular with acute to obtuse apex, margin nearly entire, rarely bluntly toothed at side, sinus V-shaped, bases rounded to cuneate, insertion line arched. ***Asexual reproduction*** by unmodified caducous leaves, lobules remain attached to the stem. ***Sexuality*** autoicous. ***Androecia*** on short branches, terminal or intercalary on branches, inflated, spicate, 515–913 µm long, 408–561 µm wide; bracts in 3–5 pairs, densely imbricate, isolobous; bracteoles occurring nearly throughout androecium, composed of 2(–3) bilobed bracteoles restricted at the base of the branch and 1–2 reduced once above. ***Gynoecia*** on short branches, with 1 subfloral innovation; bracts in one pair, subequal in size, bract lobe obovate, 526–822 µm long, 317–488 µm wide, apex rounded, incurved, margin entire, ocelli 0–14 per lobe, isolated; bract lobules lingulate, narrowly oblong to ovate, 330–442 µm long, 132–258 µm wide, 0.6–0.8 of lobe length, ca. 2/3 of the bract-lobe area, apex acute to broadly obtuse, keel slightly arched, short; bracteole shortly connate with the bracts at the base on one side or rarely on both sides, ovate to obovate, 418–587 µm long, 276–409 µm wide, apex usually emarginate or slightly bifid, lobe acute, margin entire; perianths obovate, ca. 0.5 emergent beyond bracts or sometimes almost entirely covered by bracts, 664–921 µm long, 464–624 µm wide, inflated, 5-keeled, keels crenulate or rough from projecting cells, apex usually truncate, beak short. ***Sporophytes*** not seen.

**Figure 7.**
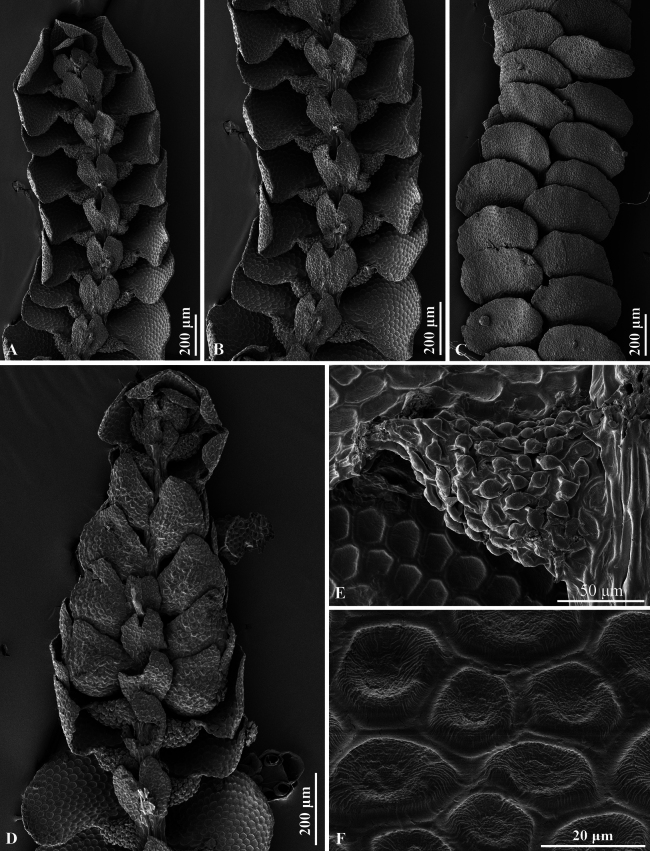
*Pycnolejeuneazhuiana* Promma & Chantanaorr. sp. nov. (SEM). **A–C.** Portions of sterile plants; **A, B.** Ventral views; **C.** Dorsal view; **D.** Androecium; **E.** Leaf lobule; **F.** Cells from middle portion of leaf, dorsal view. Photographed by C. Promma; based on *C. Promma & K. Chanakarn 20250215-30* (PSU).

##### Etymology.

The specific epithet “*zhuiana*” honours Prof. Dr. Rui-Liang Zhu of East China Normal University, Shanghai (China), who has dedicated his entire life to the study of bryophytes, particularly in the taxonomy and systematics of Lejeuneaceae and the advancement of liverwort research in Asia.

##### Distribution, habitat and ecology.

Endemic to peninsular Thailand. So far known only from its type locality at Khao Ngon Nak Mountain, Hat Noppharat Thara - Mu Ko Phi Phi National Park, Krabi Province; however, it may also occur in other areas in southern Thailand with a similar vegetation type. *Pycnolejeuneazhuiana* was found growing on tree trunks in tropical lowland forests dominated by *Baeckeafrutescens* L., *Podocarpusneriifolius* D.Don. and *Syzygiumantisepticum* (Blume) Merr. & L.M. Perry., at elevations of 380–480 m. It is often found growing in association with *Lejeuneaflava* (Sw.) Nees and *P.contigua*.

##### Taxonomic notes.

*Pycnolejeuneazhuiana* is readily distinguished by a combination of the following characters: ventral merophyte consistently 2 cells wide; oblong-ovate to oblong leaf lobes; strongly mammillose on dorsal side of leaf lobe cells; presence of 1–8 ocelli per leaf lobe which are either aggregated or isolated at the basal region of the lobe; the strongly papillate lobules with 5–6 elongate cells along the free margin; and male bracteoles occurring nearly throughout the androecial branch.

*Pycnolejeuneazhuiana* can be confused with *P.papillosa* from tropical America, which also have papillose lobues and unipapillose leaf cells. However, *P.zhuiana* is distinguished by its larger size and ovate to oblong-ovate leaf lobes, while *P.papillosa* has smaller shoots and orbicular-ovate leaf lobes. Additionally, *P.zhuiana* has 1–8 ocelli per leaf lobe, while *P.papillosa* has only 1–2(–3) ocelli per leaf lobe. *Pycnolejeuneazhuiana* also has male bracteoles almost throughout the androecial branch. In contrast, *P.papillosa* has only one male bracteole restricted to the base of the branch. The differences between the two species are shown in Table 1.

**Table 1.** Morphological differences between *Pycnolejeuneazhuiana* and *P.papillosa*. The characters of *P.papillosa* are taken from the protologues and recent publication (He 1999; Bastos et al. 2020).

Characteristic	*P.zhuiana*	*P.papillosa*
Shoot width	0.71–1.32 mm	0.6–1.0 mm
Stem in transverse section	9–11 epidermal cells, surrounding 13–15 medullary cells	7–9 epidermal cells, surrounding 9–10 medullary cells
Leaf lobe shape	Ovate to oblong-ovate, flat to slightly convex, apex plane	Orbicular-ovate, strongly convex, apex incurved
Number of ocelli per leaf lobe	1–8	1–2(–3)
Male bracteoles	nearly throughout the androecial branch, composed of 2(–3) larger bracteoles at the base and 1–2 smaller bracteoles above	only 1, restricted at the base of androecia

With regards to the male bracteole, most species of *Pycnolejeunea* have only 1 or 1–2 bracteoles per androecial branch and restricted to the base of the androecium. Except for *P.macroloba* (Nees & Mont.) Schiffn. from the Neotropics, male bracteoles are present throughout the androecium or nearly so (He 1999). However, *P.macroloba* differs from *P.zhuiana* in the larger plant (to 2.5 mm wide), the rectangular lobules (0.5–0.6 of lobe length) with 9–20 elongated cells along the lateral margin and nearly smooth lobule surface.

##### Additional specimens examined.

Thailand. Krabi: Hat Noppharat Thara - Mu Ko Phi Phi National Park, Khao Ngon Nak Mountain, 8°5.415'N, 98°46.1683'E, 380 m elev., 24 Mar 2018, *A. Senayai 64* (BKF, PSU); 8°5.2767'N, 98°46.47'E, 480 m elev., 9 Apr 2022, *S. Chantanaorrapint & A. Chantanaorrapint s.n.* (PSU); 8°5.2767'N, 98°46.47'E, 480 m elev., 15 Feb 2025, *C. Promma & K. Chanakarn 20250215-27, 20250215-28A, 20250215-29, 20250215-31* (PSU).

## Supplementary Material

XML Treatment for Pycnolejeunea contigua

XML Treatment for Pycnolejeunea grandiocellata

XML Treatment for Pycnolejeunea zhuiana

## ﻿Additional information

### Author ORCIDs

Chatchaba Promma https://orcid.org/0000-0001-5990-6251

Sahut Chantanaorrapint https://orcid.org/0000-0002-9739-0994

### Data availability

All of the data that support the findings of this study are available in the main text.
